# Predictors of Performing Function Focused Care

**DOI:** 10.1111/opn.70088

**Published:** 2026-07-11

**Authors:** Kelly Doran, Douglas Gyamfi, Siijun Zhu, Amylee Anyoha, Lauren Anderson, Abaneh Ebangwese, Barbara Resnick

**Affiliations:** ^1^ University of Maryland Baltimore, School of Nursing Baltimore Maryland USA; ^2^ University of Maryland, School of Public Health College Park Maryland USA

**Keywords:** physical activity, residents, sedentary, worksite wellness

## Abstract

**Background:**

Sedentary behaviour is highly prevalent among residents, with 84% classified as sedentary. Function Focused Care (FFC) encourages resident participation in activities to reduce inactivity. This study examined factors influencing independent living, assisted living, nursing home and memory care staff's engagement in FFC, hypothesising that staff with lower sedentary behaviours, higher physical activity and FFC knowledge, higher self‐efficacy and outcome expectations for FFC, and higher decision authority would increase FFC engagement.

**Objective:**

This study examined factors influencing independent living, assisted living, nursing home and memory care staff's engagement in FFC, hypothesising that staff with lower sedentary behaviours, higher physical activity and FFC knowledge, higher self‐efficacy and outcome expectations for FFC, and higher decision authority would increase FFC engagement.

**Methods:**

Baseline data (*n* = 139) from a randomised controlled trial were analysed in this cross‐sectional study. Staff from six facilities provided accelerometer‐measured sedentary behaviour data, as well as completed FFC behaviour observations and surveys on self‐efficacy, knowledge, decision authority and demographics.

**Results:**

The sample was predominantly female (88.2%) and African American (67.9%). Staff exhibited an average sedentary time of 11.60 h/day (SD = 2.76). Generalised linear mixed models identified that sedentary behaviour (IRR = 1.25, *p* < 0.001) was significantly associated with less FFC behaviours. Also, working as a direct care worker (IRR = 0.22, *p* = 0.002) and older age (IRR = 0.96, *p* = 0.033) were significantly associated with more FFC behaviours.

**Conclusion:**

Staff engaged in high levels of sedentary activity. Our data suggest that the risk of not performing FFC with residents increased as workers' sedentary behaviours increased.

**Implications for Practice:**

The findings from this study suggest that providing programs and encouraging staff to increase time spent in physical activity may translate to staff helping to provide residents with FFC and thereby increasing the time spent in physical activity. If these findings are reproducible, they could be used when developing staff and resident wellness programs.

## Summary

**What does this research add to existing knowledge in gerontology?:**

- Preliminary evidence suggests that increasing workers' physical activity levels may increase workers' function focused care behaviours.
- Preliminary evidence suggests that expecting staff to engage residents in function focused care may increase workers' function focused care behaviours.

**What are the implications of this new knowledge for nursing care for and with older adults?:**

- These findings suggest workers' personal physical activity behaviours could influence residents' engagement in physical activity.
- These findings suggest providing workers with expectations to engage residents in function focused care behaviours could influence residents' physical activity levels.

**How could the findings be used to influence practice, education, research, and policy?:**

- If reproducible, these findings could suggest combining staff and resident physical activity programs could be beneficial for both groups.

## 1 Introduction

The average US adult spends 9.5 waking hours a day sedentary, with sedentary behaviour typically increasing with age (Matthews et al. 2021). Sedentary behaviour is negatively associated with unhealthy behaviours such as poor sleep quality and poor social well‐being (Chen et al. 2021; You et al. 2023), a variety of chronic diseases such as cancer, cardiovascular disease, diabetes (Matthews et al. 2012; Park et al. 2020), as well as a variety of mental health outcomes such as depressive symptoms, anxiety, suicidal ideation and burnout symptoms (del Pozo Cruz et al. 2020; L. Jiang et al. 2020; Mincarone et al. 2024). Overall sedentary behaviour has been linked to early death (Matthews et al. 2012; Rojer et al. 2020). Specifically, approximately 8% of deaths nationwide are attributed to sedentary behaviour and/or lack of physical activity (Carlson et al. 2018).

The consequences of sedentary behaviour call for action, which Healthy People 2030 has responded to with more than 25 objectives connected to physical activity (Office of Disease Prevention and Health Promotion, n.d.). One strategy to increase physical activity that has been endorsed by the Centers for Disease Control and Prevention is exercise prevention programs delivered at worksites (Centers for Disease Control and Prevention 2025). However, it has been well documented that physical activity relapse is common, and sustaining regular physical activity is challenging, with dropout rates from exercise programs as high as 80% (Baillot et al. 2021; Marcus et al. 2000), suggesting innovative and tailored physical activity programs are needed (Baillot et al. 2021; Collado‐Mateo et al. 2021).

### 1.1 Sedentary Behaviour in Older Adults

In addition to the consequences of sedentary behaviour noted above, there are additional concerns for sedentary older adults, such as decline in cognitive function, falls and reduced function and/or disability (Coelho et al. 2020; Jiang et al. 2022; Saunders et al. 2020; You et al. 2023). This is important given 84% of residents in assisted living are classified as sedentary based on objective measurement (Resnick et al. 2021). Long‐term care residents with higher acuity (e.g., acute exacerbations of underlying disease such as acute heart failure or advanced memory issues) are more sedentary than their counterparts (Leung et al. 2021).

One way to reduce sedentary behaviour in older adults is function focused care (FFC). Function focused care is a philosophy of care in which staff encourage residents to participate in all care activities (Resnick et al. 2021; Resnick, Simpson, Bercovitz, et al. 2006). Examples of FFC include having a resident participate in bathing and dressing by role modelling the behaviour for the resident; walking a resident to the bathroom versus using a commode chair or bedpan; and walking a resident to the dining room versus pushing them in a wheelchair. Participation in FFC has been shown to increase residents' physical activity levels and improve other outcomes associated with a reduction in sedentary behaviour, such as function, falls and mental health (Gruber‐Baldini et al. 2011; Resnick et al. 2011, 2021; Resnick, Wells, et al. 2016; Resnick and Galik 2013).

There are some challenges associated with getting staff to provide FFC such as: lack of support from staff; lack of staff knowledge; lack of worker autonomy on how care is delivered and staff's' low self‐efficacy and outcome expectations for physical activity and/or their lack of engagement in physical activity (Resnick et al. 2008, 2022; Resnick, Simpson, Galik, et al. 2006). However, to facilitate encouragement of FFC, Social Cognitive Theory‐based interventions can be used to guide interpersonal interactions between residents and increase resident physical activity levels (Resnick et al. 2008, 2022; Resnick, Simpson, Galik, et al. 2006). Social Cognitive Theory includes two main concepts: self‐efficacy expectations and outcome expectations and suggests that the stronger the individual's self‐efficacy expectations and outcome expectations are the more likely it is that the person will initiate and persist with an activity. Self‐efficacy expectations are defined as the belief that one is able to execute a course of action to achieve a specific goal and are typically influenced by four sources. The four sources are: enactive mastery (engaging in the behaviour), vicarious experience (hearing peers talk about their engagement in the behaviour), verbal persuasion (a peer providing encouragement to engage in the behaviour) and physiological arousal (enhancing/highlighting positive aspects of the behaviour and minimising or finding solutions for any negative aspects of the behaviour). Outcomes expectations are defined as the beliefs that if a certain behaviour is performed, it will lead to an anticipated outcome that is meaningful to the person. Ideally, all four sources of self‐efficacy expectations are activated, and outcome expectations are evoked to enlist behaviour change (Bandura and Watts 1996; Resnick 2008).

The objective of this study was to understand what factors influence staff's use of FFC behaviours with residents residing in independent living, assisted living, nursing homes and memory care facilities. Prior research has noted that healthcare workers who engage in physical activity are more likely to encourage residents to engage in physical activity (Borges et al. 2024). Therefore, we hypothesised that lower levels of sedentary behaviour among staff would result in increased FFC behaviours with residents. We also hypothesised that higher staff beliefs around self‐efficacy and outcome expectations as well as increased knowledge about FFC, would be associated with providing residents with FFC and encouragement to engage in physical activity (Bandura and Watts 1996; Fredriksson et al. 2018; Resnick, Beaupre, et al. 2016; Wafi et al. 2024). Lastly, given prior work has suggested that a lack of autonomy over how work is conducted can serve as a barrier for FFC (Resnick et al. 2008, 2022), we hypothesised that increased autonomy over work tasks would result in increased engagement in FFC with residents. Therefore, in summary, we hypothesised that staff who had: lower levels of sedentary behaviours, increased physical activity and FFC knowledge, higher self‐efficacy and outcome expectations for FFC as well as higher discretion over how they performed work tasks (i.e., decision authority) would engage in more FFC activities.

## 2 Methods

### 2.1 Design

This was a secondary data analysis using a cross‐sectional design. The study used baseline data from a 12‐month randomised controlled trial titled the Worksite Heart Health Improvement Project + Function Focused Care (WHHIP+FFC, clinicaltrials.gov ID: NCT04166643). The parent study sought to reduce organisational sources of job stress within LTC settings, improve heart‐healthy behaviours (e.g., physical activity) among staff and encourage staff to engage residents in physical activity. The study was conducted in six facilities that offered independent living, assisted living, nursing homes and/or memory care within one state on the East coast of the United States. Baseline data used for this study were collected from July 2022 to Aug 2023.

### 2.2 Sample

Staff learned about the study via flyers, ‘meet and greet’ sessions and discussions at staff meetings. The parent study's eligibility criteria stated staff must be: at least 18 years of age, employed by the facility and able to read and write English. Participants were excluded if they could not pass the Evaluation to Sign Consent test (Resnick et al. 2007), which tests participants' understanding of key consent aspects (i.e., study activities; withdrawing process; adverse event reporting; how sites were randomised). The study was approved by University of Maryland Baltimore's Institutional Review Board (#HP‐00086939) and participants signed a consent form before participating in study activities. Across the six LTC facilities, 373 staff (47% of the total number of staff) talked with a member of the research team about the study. Of these 373 potential participants, 139 (37%) consented for the parent study. All participants were included in this secondary data analysis.

### 2.3 Measures

In addition to answering demographic questions, participants completed a survey, were observed during patient care interactions and wore an accelerometer. Participants were encouraged to complete a survey during their workday. The survey could be completed via paper and pencil or electronically, based on their preference. The six‐item validated Nursing Assistant's Self‐Efficacy for Restorative Care (Resnick et al. 2008) scale was used to assess confidence in performing FFC activities. The scale ranges from 6 to 30, with higher scores indicating stronger self‐efficacy. The six‐item validated Nursing Assistants' Outcome Expectation for Restorative Care (NAOERC) (Resnick et al. 2008) was used to assess beliefs in the outcomes associated with performing FFC activities. The scale ranges from 6 to 30, with a higher score indicating stronger outcome expectations. The valid nine‐item decision authority subscale from the Job Strain Model tool (Bosma et al. 1997; Kivimäki et al. 2004) was used to assess a subset of job control—the ability to make choices around work tasks. The scale ranges from 9 to 36, with a lower score indicating greater decision authority. Lastly, the survey included three questions to assess participants' physical activity knowledge and two questions to assess FFC knowledge. The first physical activity knowledge question asked participants to write how many minutes of physical activity were required each week (Centers for Disease Control and Prevention 2023). If they wrote that they were unaware or unsure of the guidelines or got the number of minutes wrong, there were coded as incorrect. The remaining two physical activity questions used a multiple‐choice format to ask participants to define moderate and vigorous activity using the talk‐test criteria (Centers for Disease Control and Prevention 2023). For FFC knowledge, participants were asked two multiple‐choice questions about the purpose of FFC and how to engage in FFC. These individual items were dichotomised as either correct or incorrect and then summed to create a composite physical activity and FFC knowledge score. Higher scores indicated more FFC and physical activity knowledge.

The valid FFC Checklist for Caregivers (Resnick et al. 2021) assesses FFC interactions. Research staff observed workers for 30 min to complete the FFC checklist during routine work tasks and recorded performed, not performed or not observed for 19 specific care behaviours. By looking at the opportunities staff had to engage in FFC and how much they did engage in FFC, you can calculate the percent staff who engaged in FFC. For the analysis, we used the FFC not‐performed count. This variable is calculated by summing the number of not‐performed behaviours. The count could range from 0 to 19, with a higher score indicating more not‐performed behaviours.

Physical activity and sedentary behaviour were measured using an accelerometer‐ the MotionWatch 8 (CamNtech Ltd 2025). The MotionWatch 8 is a validated accelerometer widely used for assessing physical activity levels in healthy older adults (Chakravarthy and Resnick 2017; Landry et al. 2015). Participants were encouraged to wear the MotionWatch for 5 days with days 2, 3 and 4 used for analyses and days 1 and 5 for placement and removal (You et al. 2023). The watch was set to triaxial mode with 60 s epochs. Although for the purposes of this secondary data analysis, data from only full days of physical activity data were included (e.g., days 2–4). Participants' activity levels were determined based on established cut points (Landry et al. 2015); sedentary activity: < 178.51 counts per minute (cpm), light activity: 178.5 cpm—562.5 cpm, moderate activity: > 562.5 cpm and vigorous activity: > 1020 cpm. Determining cut points for a sample is challenging, given the limited established outpoints (Chakravarthy and Resnick 2017). The cut points suggested by Landry et al. (2015) were used as their sample was the closest match to our sample, and their device settings were closest to the settings used in this study. Minutes per physical activity were then recorded.

### 2.4 Data Analysis

SPSS version 28 and 29 (IBM 2025) and Stata 18 (StataCorp. 2024) were used to conduct the analysis and a *p* < 0.05 was deemed significant. Descriptive statistics such as frequencies and mean (SD) were used to describe the data. Since the outcome variables were the count variables, generalised linear mixed models (GLMM) with a Poisson distribution were used to test our hypothesis. The random intercept of site was included to account for clustering within sites. The fixed effects included sedentary time (main predictor) and decision authority, self‐efficacy and outcome expectation for FFC, physical activity knowledge, FFC knowledge, as well as demographics such as age, years of employment and direct care worker status. There is no indication of multicollinearity among the covariates since the variance inflation factors were less than 1.8.

Maximum likelihood estimation was used in the generalised linear mixed models to address the missing data (8% for sedentary time; approximately 17% for decision authority, FFC and PA knowledge, direct care worker status and years of employment; about 27% for FFC outcome expectation and 37% self‐efficacy). Missing‐data analysis was performed. The Little's missing at completely random (MCAR) test (*p* < 0.001) showed missing not MCAR. We then conducted covariate‐dependent missingness (CDM) tests including age, direct care worker status and years of employment as covariates, extending Little's missing completely at random test. Since CDM tests were non‐significant (*p* = 0.998), the missing‐data patterns were consistent with a covariate‐dependent missingness mechanism, which is compatible with a missing‐at‐random (MAR) assumption (Li 2013). As a sensitivity test, we conducted multiple imputation in chain's equation (MICE); the results were consistent with those from ML estimation in GLMMs. Therefore, we reported the results from the ML estimate.

## 3 Results

The study included a total of 139 participants and their demographics are described in Table 1. Almost 38% of the sample provided direct care to residents including nurses, nursing assistants, medication technician or care coordination workers. The remaining sample was from a variety of positions such as managers (21.2%), housekeeping (10.6%), dietary (8.0%), activities (2.7%), concierge (7.1), business administration (10.6%) and facilities (1.9%). The majority of the sample worked day shift, with 19.5% of participants reporting evening shift work, 9.7% reporting they worked rotating shifts and 3.5% reporting night shift as their primary assignment. The most common race reported was African American/Black (67.9%), followed by Caucasian (28.2%).

**TABLE 1:** Baseline variables *n* = 139.

Variable	*N*	%
Female	119	88.2
Educational level up to High school	41	30.8
Black or African American	89	67.9
Worked Primarily Day Shift	76	67.3
Direct care Worker	43	38.1
Primarily works 8 h shift	83	72.2

	Mean	SD
Age (years)	50.1	134
Tenure (years)	8.1	7.4
Physical activity knowledge	1.28	0.92
FFC knowledge	0.88	0.78
Average daily sedentary time (hours)	11.59	2.76
Decision Authority	20.1	5.5
Self‐efficacy expectations for FFC	20.9	6.0
Outcome expectations for FFC	22.6	6.1
Percent engaged in FFC	91.6	21.0

Abbreviation: FFC, Function Focused Care.

Total knowledge scores are reported in Table 1. Regarding knowledge of the amount of weekly time required for physical activity (Centers for Disease Control and Prevention 2023), only 9.6% of participants were able to articulate 150 min per week. When using the talk‐test criteria (Centers for Disease Control and Prevention 2023), only 54.9% of participants and 67.3% of participants were able to define moderate and vigorous activities correctly, respectively. More than half of the participants were able to correctly define the purpose of FFC (42.4%) and how to engage in FFC (45.3%).

The accelerometer data showed that sedentary or light‐intensity activity was the most common behaviours. The mean sedentary time was 11.60 (SD = 2.76) hours with a minimum amount of 6.26 h and a maximum of 23.5 h. For light‐intensity physical activity, the mean daily time among participants was 12.26 (SD = 2.71) hours with a minimum of 2.71 h and a maximum of 17.73 h. The median daily time spent in moderate physical activity was 0.00 min with a range from 0.00 min to 2.13 h. Per the actigraphy data, no participant engaged in vigorous activity.

As shown in Table 2, based on GLMM for every hour of increase in sedentary activity, the IRR of not providing FFC increased by 25% (IRR = 1.25, *p* < 0.001). This suggests that as workers' sedentary behaviour increased, workers engaged in fewer FFC behaviours. The model also showed there was a decrease in the percentage of time that direct care workers did not perform FFC with residents (78%, *p* = 0.002) when compared to those not in direct care roles. This suggests that direct care workers engage in more FFC than non‐direct care workers. Age was associated with FFC performance in the multiple analysis (IRR = 0.96, *p* = 0.033), indicating older staff tend to engage in more FFC behaviours.

**TABLE 2:** Summary of regression analysis for FFC not performed, *n* = 139.

Variable	Bivariate analysis		Multiple analysis	
	IRR(95% CI)	*p*	IRR(95% CI)	*p*
Average sedentary time, hour	1.17 (1.11, 1.23)	< 0.001	1.25 (1.14, 1.37)	< 0.001
Decision authority	0.97 (0.93, 1.02)	0.306	0.96 (0.87, 1.07)	0.507
Self‐efficacy for FFC	0.96 (0.91, 1.01)	0.1	0.94 (0.82, 1.07)	0.335
Outcome expectations for FFC	0.96 (0.92, 1.01)	0.109	1.08 (0.95, 1.24)	0.234
Physical activity knowledge	1.78 (1.31, 2.4)	< 0.001	1.83 (0.91, 3.68)	0.093
FFC knowledge	0.74 (0.51, 1.07)	0.109	0.56 (0.23, 1.37)	0.204
Years employed	1.0 (0.99, 1.0)	0.089	1 (1, 1.01)	0.463
Direct care worker	0.24 (0.12, 0.48)	< 0.001	0.22 (0.08, 0.59)	0.002
Age	0.99 (0.98, 1.01)	0.441	0.96 (0.92, 1)	0.033

*Note:* Multiple analysis was done with all of the variables in the table included in the model.

Abbreviations: 95% CI, 95% confident interval; IRR, incident rate ratio.

## 4 Discussion

Our hypothesis that self‐efficacy for FFC, outcome expectations for FFC, decision authority, physical activity knowledge and FFC knowledge influenced FFC engagement was not supported. It is possible that the lack of association between self‐efficacy and outcome expectations for FFC and engagement in FFC activities may, in part, be explained by a lack of knowledge. Past work with this population suggests low physical activity knowledge, as seen with our sample, may cause over‐ and underestimation of self‐efficacy and outcome expectations (Flannery, Resnick, and McMullen 2012). Self‐efficacy and outcome expectations for FFC may also not have been significant, as they were both skewed and could have been influenced by social desirability bias. It is possible that physical activity and FFC knowledge were not significant, as knowledge may be foundational for behaviour change, but it alone is usually not sufficient to change behaviour. Future work may wish to explore the impact of knowledge, along with skills to execute the behaviour and internal and external motivators (Heimlich and Ardoin 2008; Resnick et al. 2008; Resnick, Simpson, Galik, et al. 2006).

Our study found that sedentary behaviour was the only factor that was significantly associated with staff engaging in FFC activities. We found that as sedentary behaviour increased, the risk of not performing FFC behaviours increased. This is in line with prior work suggesting healthcare providers who engage in physical activity are more likely to encourage patients/residents to engage in exercise (Lee et al. 2024). Additionally, prior work has suggested that up to 70% of patients/residents are more likely to follow recommendations to engage in physical activity if their provider encourages it and if the patient/resident believes the healthcare provider making the recommendation also engages in physical activity themselves (Lobelo and de Quevedo 2014). Our findings are in line with other work suggesting physical activity interventions with healthcare workers could not only increase healthcare provider engagement in physical activity but also possibly increase patient/resident physical activity levels by increasing resident engagement in FFC activities (Chappel et al. 2023; Doran, Resnick, Zhu, and Alghzawi 2018; Doran and Resnick 2022; Lobelo and de Quevedo 2014). The finding that the risk of not performing FFC behaviours was influenced by job role and age is not surprising. Direct care workers typically have more care interactions with residents and, therefore, are more likely to have been exposed to FFC training as well as have an increase in confidence and skills to perform FFC behaviours. This would also be true for staff who have more experience in this profession.

Our findings suggest low levels of physical activity, as well as low physical activity and FFC knowledge in this population. It has been consistently documented that healthcare providers often do not meet physical activity guidelines and/or engage in mainly sedentary behaviour (Chappel et al. 2023; Doran and Resnick 2017; Flannery, Resnick, et al. 2014; Hajo et al. 2020; Schult et al. 2011) with some reports suggesting they might engage in less physical activity than the general population (Doran and Resnick 2017; Flannery, Resnick, et al. 2014; Schult et al. 2011). Prior pilot work looking at healthcare workers suggests that, on average, 73% of an eight‐hour shift is spent in sedentary behaviour when measured objectively, with most of the remaining time being classified as light activity (Chappel et al. 2017, 2023). It has been suggested that the lack of adherence to physical activity guidelines among healthcare workers may in part be due to their lack of understanding (Flannery, Burket, and Resnick 2014). These prior findings are supported by our results, given that about 9% of participants were able to articulate physical activity guidelines, and the average daily time for sedentary behaviour was over 11 h a day, while the majority of the remaining time was spent in light activity.

Our findings are interesting, and if replicated it may suggest that increasing time in physical activity may facilitate an increase in FFC behaviours being provided to residents during care interactions. Worksite wellness programs that include physical activity components with workers in long‐term care settings have demonstrated improvements in health outcomes (e.g., blood pressure), work outcomes (e.g., work ability), mood and health behaviours (e.g., sleep) (Doran, Resnick, Alghzawi, and Zhu 2018; Flannery, Resnick, and McMullen 2012; Flannery, Resnick, Galik, et al. 2012). Additionally, upon follow‐up after the completion of a worksite wellness program with long‐term care workers, they reported engaging residents in wellness behaviours (e.g., exercise) as a result of the wellness program (Doran, Resnick, Zhu, and Alghzawi 2018). Therefore, if our findings are replicated to maximise benefits, future work may wish to explore the benefits of combining resident and staff physical activity programs to explore any synergistic and spillover effects (Flannery and Resnick 2014). While worksite wellness programs have advocated for inclusion of the entire facility (i.e., both direct care and non‐direct care staff) to help facilitate a culture of wellness (Bradley et al. 2016; Sorensen et al. 2011), our study found direct care workers engaged in more FFC behaviours. While not as often as direct care staff, non‐direct care staff in LTC facilities have opportunities to engage residents in physical activity (Paudel et al. 2020). Therefore, if these findings are replicated, future work may wish to explore if non‐direct care staff need training in providing FFC interventions to residents.

## 5 Limitations

The findings from this study are limited by the sample size, which limits our generalisability and estimation ability. Further limiting generalisability is that our sample included a small number of communities from a single East Coast state within the United States. Due to low levels of moderate and vigorous activity in our sample, the analysis focused only on sedentary data. While accelerometers are considered the gold standard for physical activity measurement, they might not capture all physical activity (Chappel et al. 2023) and it is possible that the use of different cut points such as those provided by Freedson (Freedson et al. 1998) may have resulted in different findings. Forty percent of our sample were non‐direct care staff and while the research team attempted to collect FFC observations while participants were engaging with residents it is unclear how much engagement non‐direct care staff had providing care for residents. However, several non‐direct care jobs (e.g., activities, dietary, etc.) have important relationships with residents (Paudel, et al. 2022) and are able to encourage FFC through their job tasks (e.g., a dietary staff member encouraging a resident to walk to meals and an activity staff member providing activities that incorporate physical activity). Lastly, in addition to the above reasons for the lack of influence of knowledge, measurement bias could also have influenced our results. Both the physical activity and FFC knowledge scores were intended to assess treatment fidelity of the parent study. Thus, it is possible that they did not comprehensively assess important factors of knowledge. For example, while assessing knowledge of physical activity guidelines is a common assessment of physical activity knowledge, this study failed to assess other factors, such as impact on health, which might be important (Fredriksson et al. 2018). Similarly, the FFC measures failed to assess important factors such as workers' knowledge around safety implementing FFC (e.g., preventing falls), which has been reported as an FFC implementation barrier in the past (Resnick et al. 2008; Resnick, Simpson, Galik, et al. 2006).

## 6 Conclusion

Our study aimed to investigate the factors that predict staff's participation in FFC behaviours. Our findings provide preliminary evidence to suggest staff's' physical activity levels are associated with engaging residents in FFC behaviours. This might suggest that targeting staff physical activity and/or including staff and residents in physical activity interventions together may benefit both groups. Future work may wish to replicate these findings to explore whether addressing sedentary behaviour in staff could improve the health of both staff and residents.

### 6.1 Significance/Implications

These pilot findings could be used to guide other studies to explore the importance of workers' sedentary behaviour in encouraging FFC behaviours with residents. If these findings are reproducible, they could be used when developing staff and resident wellness programs.

## Author Contributions

**Kelly Doran:** conceptualisation; methodology, investigation, writing – original, supervision, project administration and funding acquisition; **Douglas Gyamfi:** formal analysis, data curation, writing – original and visualisation; **Siijun Zhu:** conceptualisation; methodology, investigation, writing – original, formal analysis, data curation, visualisation and funding acquisition; **Amylee Anyoha:** writing – original, investigation and project administration; **Lauren Anderson:** data curation, writing – original, investigation and project administration; **Abaneh Ebangwese:** data curation, writing – original, investigation and project administration; **Barbara Resnick:** conceptualisation; methodology, writing – review and editing and funding acquisition.

## Funding

This work was funded by the American Heart Association (grant # 19TPA34830011), the University of Maryland School of Nursing and the Building Healthy Behaviours Across the Lifespan research centre.

## Ethics Statement

This study was approved by University of Maryland Baltimore's IRB (#HP‐00086939).

## Consent

Participants signed consent before participating in the study.

## Conflicts of Interest

The American Heart Association provided the authors (K.D., B.R., S.Z.) the resources and time (e.g., salary coverage) to complete the project discussed as well as this manuscript. The American Heart Association provided funds to the University of Maryland and not the authors directly. The authors report no other conflicts of interest.

## Data Availability

The data that support the findings of this study are openly available in openICPSR at https://www.openicpsr.org/openicpsr/project/244985/version/V1/view, reference number https://doi.org/10.3886/E244985V1.
